# Computerized Assessment of Communication for Cognitive Stimulation for People with Cognitive Decline Using Spectral-Distortion Measures and Phylogenetic Inference

**DOI:** 10.1371/journal.pone.0118739

**Published:** 2015-03-24

**Authors:** Tuan D. Pham, Mayumi Oyama-Higa, Cong-Thang Truong, Kazushi Okamoto, Terufumi Futaba, Shigeru Kanemoto, Masahide Sugiyama, Lisa Lampe

**Affiliations:** 1 Aizu Research Cluster for Medical Engineering and Informatics, Center for Advanced Information Science and Technology, The University of Aizu, Aizu-Wakamatsu, Japan; 2 Chaos Technology Research Lab, Shiga, Japan; 3 School of Computer Science and Engineering, The University of Aizu, Aizu-Wakamatsu, Japan; 4 School of Nursing and Health, Aichi Prefectural University, Aichi, Japan; 5 Faculty of Intercultural Communication, Ryukoku University, Shiga, Japan; 6 Discipline of Psychiatry, Sydney Medical School, The University of Sydney, Sydney, Australia; Shenzhen institutes of advanced technology, CHINA

## Abstract

Therapeutic communication and interpersonal relationships in care homes can help people to improve their mental wellbeing. Assessment of the efficacy of these dynamic and complex processes are necessary for psychosocial planning and management. This paper presents a pilot application of photoplethysmography in synchronized physiological measurements of communications between the care-giver and people with dementia. Signal-based evaluations of the therapy can be carried out using the measures of spectral distortion and the inference of phylogenetic trees. The proposed computational models can be of assistance and cost-effectiveness in caring for and monitoring people with cognitive decline.

## Introduction

Communication is a complex and dynamic process to comprehend words or signals between two or more participants. The ability to communicate with people whose speech or hearing is impaired by cognitive decline is a skill that can be developed over time with practice. Care-givers use communication skills to provide individuals with professional care, establish supportive relationships, obtain information, and assist with changing behavior. Thus, therapeutic communication is a basis of the nurse-client relationship [1].

A decline in cognitive ability severe enough to interfere with daily life is seen in dementia, which is caused by damage to brain cells. In addition to drug treatments, there are other interventions that can treat or manage the symptoms of dementia. These include a range of therapies such as talking therapies, reminiscence therapy, cognitive stimulation therapy and complementary therapies. In particular, talking therapies may also help with dementia associated with depression or anxiety (http://www.alzheimers.org.uk). Effective communication can improve the quality of life for individuals with dementia. However, it is of note that many people with dementia do not have the opportunity for conversation and expression of their feelings about their own life [2] in addition to the basic services they are provided [3]. This is because cognitive impairment in people with dementia reduces their ability to communicate effectively, which in turn adversely affects the ability of the care-giver to identify their needs [4]. Research indicates that people with dementia are concerned with these two issues, and that effective communication takes time [3]–[6].

Several therapeutic interventions have been developed to work directly with people with dementia on an individual or group basis, and also indirectly with family and professional care-givers and social-care professionals to improve communication and quality of life for people with dementia [7]. In fact, skills needed for effective communication with people with dementia have been explored, and include factors that influence the communication process and therapeutic relationships between nurses and patients [4]. Communication in the form of encouraging talking, body language, physical contact, and active listening is thought to be fundamental to the provision of good dementia care. Furthermore, symptoms of depression and anxiety are common in people with dementia and mild cognitive impairment. Although treatment of these symptoms is widely recommended in guidelines, the best way to carry out the treatment is still not clear. While drugs are thought to have limited effectiveness in this context and may involve the risk of side effects, psychological treatments can be applied as an alternative to improve the mental wellbeing and cognitive function of people with cognitive impairment [8]. While traditional cognitive training interventions are delivered by humans, a recent review concluded that computer-based cognitive interventions are comparable or better than paper-and-pencil cognitive training approaches [9]. This review suggests that the utilization of computerized technology offers an effective and labor-saving method for improving and maintaining the quality of life and confidence of the individual with age-related impairment in cognitive function.

Given the prevalence and corresponding monetary costs of dementia on the public health system, it was pointed out that the need for the assessment of cognitive intervention techniques in terms of cost-efficiency is critical, and cognitive stimulation therapy can be effective and more cost-effective than conventional treatment [10, 11]. However, little effort has been made to investigate the important issue of evaluating this type of psychological intervention [12]. As a consequence, there is currently no agreement in place for the definition of cognitive intervention or the measure of its success [13]. These factors are necessary to ensure the efficacy of cognitive stimulation and intervention design [14].

With the advanced development of electronic physiological technology, photoplethysmography (PPG) has been applied as a low-cost physiological measurement of pulse waves for studying age-related health conditions [15]–[20]. This paper presents a pilot study of synchronized PPG-based evaluation of interpersonal communication as an intervention for stimulating the cognitive function of individuals with decreased cognitive ability, more specifically, the study involves elderly people with dementia. Interpersonal communication is the face-to-face process of exchanging information and feelings through verbal and non-verbal messages. It is not just about what is actually said, but how the information is expressed and how the non-verbal messages communicated by means of vocal tone, facial expressions, gestures and body language. It has been realized that physiological information changes in response to stress, including behaviors and emotions, can be evaluated through proxy autonomic measures such as finger pulse rate [21]; and finger PPG, which is a simple and non-invasive technology used to monitor peripheral circulation for assessing mental construct [22], can be used to measure vital changes in the body for detecting how an individual performs, feels, and responses [21]. Furthermore, it was reported that stress can be detected with the increase of finger pulse rate and decrease of pulse wave amplitude [20]; and finger PPG can be used to capture, with great precision, the immediate physiological response to a stimulus [23]. Because this PPG-based evaluation for cognitive intervention is a signal-based approach, the assessment of the influence of the care-giver on the participant can be analytically evaluated using spectral-distortion measures, which has been found useful for analysis of biological data [24], and the inference of phylogenetic tree reconstruction methods. Using these computational models for cognitive stimulation therapy assessment, the research suggests that the proposed therapeutic assessment is economically feasible. Experimental results are reproducible. Such reproducibility of intervention assessments can also be helpful for reaching agreement among experts in the presence of uncertainty around standardization of the course and outcome of cognitive stimulation therapy for cognitive impairment.

## Methods

### Participants

The study was carried out in Shosha Himawari (Japan) care home, and involved 48 participants (5 males and 43 females). After receiving an explanation about the study, those who understood the purpose of the study and agreed to participate were asked to sign the written informed consent form in the presence of the care-home manager. When a participant was considered incapable of providing consent, the consent form was signed by either a family member of or the nurse caring for that particular participant. This study was approved by the Himeji Himawari Nursing Home Ethics Committee.

The mean age of the participants was 85.23 years (standard deviation = 6.93 years, and range = 66–100 years). These elderly participants were clinically diagnosed with dementia. There are 5 grades ranging from 1 to 5 (the higher the more severe), indicating the severity of dementia evaluated by a geriatric psychiatrist. The grades of these 48 participants were from 1 to 4 (mean = 3.16, and standard deviation = 0.69). There are 5 levels of care given by the care home, designating the intensity of professional care that clients require for their needs. The participants involved all 5 levels of care (mean = 3, and standard deviation = 1.5) at the time of recordings of their finger pulse waves (finger PPG). The qualified care-giver, was a female familiar with the daily living activities of the participants, who was 45 years old at the time of the experimental PPG measurement.

### Synchronized PPG measurement and content of cognitive stimulation communication

The participants’ pulses of the index finger of the left hand were measured with a PPG sensor connected to a personal computer for 3 minutes before the therapeutic session, 3 minutes during the one-on-one session with the care-giver whose left-finger pulse waves were also recorded at the same time as each of the participants, and 3 minutes after the session. The PPG measurements of the participants before and after the session were designed to be used as the control signals to study the influence of the care-giver over the participants during the cognitive stimulation therapy. The therapeutic conversation mainly includes questions about the participants’ feelings, physical condition, what they had done before the measurement, hobbies, family, friends, and memories of the past. The cognitive stimulation therapy between the care-giver and elderly participants involved conversation only (without touching) by engaging the participants in talking about topics of relevance to them.

### Spectral-distortion measures

Before the calculation of the spectral distortion between the finger pulse waves of the care-giver and each of the participants, which indicates the degree of matching between the pair of people during the cognitive-function stimulating therapy, the pre-processing of the PPG signals were carried out to remove the trend (baseline shift resulting from sensor drift) to represent the true amplitude of the pulse waves by fitting a low order polynomial (degree 6 was used in this study) to the signal for detrending (by subtracting the value of the polynomial from the signal), and smoothed by using the Savitzky-Golay filter [25].

Spectral distortion measures are designed to compute the dissimilarity or distance between two (power) spectra [26] (the power spectrum of a signal describes how the variance of the data is distributed over the frequency components into which the signal may be decomposed, and the most common way of generating a power spectrum is by using a discrete Fourier transform) of the two feature vectors, originally developed for comparison of speech patterns [27]. Three methods of spectral-distortion measures were used in this study, based on their popular applications in signal processing: Itakura distortion (ID), log spectral distortion (LSD), and weighted cepstral distortion (WCD) [27]. Unlike the Itakura distortion, both log spectral distortion (distance) and weighted cepstral distortion (distance) are symmetric.

Consider two signals *S* and *S*′, and their two spectral representations *S*(*ω*) and *S*′(*ω*), respectively, where *ω* is normalized frequency ranging from −*π* to *π*.

The Itakura-Saito distortion (ISD) between *S* and *S*′ is defined as [28]

$$\begin{array}{c} ISD\left(S,S^{\prime }\right)=\int_{-\pi }^{\pi }\left[\frac{{|S\left(\omega \right)|}^{2}}{|S^{\prime }{\left(\omega \right)|}^{2}}+\log\frac{|S^{\prime }{\left(\omega \right)|}^{2}}{{|S\left(\omega \right)|}^{2}}-1\right]\frac{d\omega }{2\pi }, \end{array}$$ (1)

where

$$\begin{array}{c} {|S\left(\omega \right)|}^{2}=\frac{\sigma^{2}}{|1+a_{1}e^{-j\omega }+a_{2}e^{-j2\omega }+\cdots +a_{p}e^{-jp\omega }{|}^{2}}, \end{array}$$ (2)

where *σ* and *a*_*i*_, *i* = 1, …, *p*, are the gain and *i*th linear-predictive-coding coefficients of the *p*th-order LPC model [27], respectively (in digital signal processing, linear prediction is often called linear predictive coding (LPC) that estimates future values of a discrete-time signal as a linear function of previous samples, used for representing the spectral envelope of a digital signal in a compressed form).

$$\begin{array}{ccc} ID(S,S^{\prime }) & = & \min_{\sigma^{\prime }>0}ISD\;\left(\frac{\sigma^{2}}{{|A\left(\omega \right)|}^{2}},\frac{{\sigma^{\prime }}^{2}}{|A^{\prime }{\left(\omega \right)|}^{2}}\right) \end{array}$$ (3)

$$\begin{array}{ccc} & = & \log\int_{-\pi }^{\pi }\frac{|1+a_{1}^{\prime }e^{-j\omega }+a_{2}^{\prime }e^{-j2\omega }+\cdots +a_{p}^{\prime }e^{-jp\omega }{|}^{2}}{|1+a_{1}e^{-j\omega }+a_{2}e^{-j2\omega }+\cdots +a_{p}e^{-jp\omega }{|}^{2}}\frac{d\omega }{2\pi }, \end{array}$$ (4)

where $\frac{\sigma^{2}}{|A(\omega ){|}^{2}}$ and $\frac{{\sigma^{\prime }}^{2}}{|A^{\prime }(\omega ){|}^{2}}$ are two LPC spectra of two given autoregressive models of *S*(*ω*) and *S*′(*ω*), respectively.

The distortion defined in Eq. (4) is known as the Itakura distortion. It is also known as the log-likelihood ratio distortion or the gained-optimized Itakura-Saito distortion that can be derived as follows [29, 30]:

$$\begin{array}{c} ID\left(S,S^{\prime }\right)=\log\frac{a^{T}R^{\prime }a}{\sigma^{\prime 2}}, \end{array}$$ (5)

where *σ*′^2^ is the prediction error of *S*′ produced by the linear predictive coding (LPC) [27], **a** is the vector of LPC coefficients of *S*, **R**′ the LPC autocorrelation matrix of *S*′. It is shown that *ID*(*S*, *S*′) ≠ *ID*(*S*′, *S*), hence to make the measure symmetrical, a natural expression of its symmetrized version, denoted as *ID*_*s*_(*S*, *S*′), is

$$\begin{array}{c} ID_{s}\left(S,S^{\prime }\right)=\frac{ID\left(S,S^{\prime }\right)+ID\left(S^{\prime },S\right)}{2}. \end{array}$$ (6)

The log spectral distortion distance between two signals *S* and *S*′ is defined as [27]

$$\begin{array}{c} LSD\left(S,S^{\prime }\right)=\int_{-\pi }^{\pi }{|V\left(\omega \right)|}^{m}\;\frac{d\omega }{2\pi }, \end{array}$$ (7)

where *m* = 1 gives the mean absolute log spectral distortion, *m* = 2 defines the root-mean-square log spectral distortion that has been widely applied in speech signal processing and also used in this study, when *m* approaches ∞ Eq. (7) reduces to the peak log spectral distortion, and *V*(*ω*) is the difference between the two spectra *S*(*ω*) and *S*′(*ω*) on a log magnitude versus frequency scale and defined by

$$\begin{array}{c} V\left(\omega \right)=\log S\left(\omega \right)-\log S^{\prime }\left(\omega \right). \end{array}$$ (8)

The weighted cepstral distortion between *S* and *S*′ is defined by [27]

$$\begin{array}{c} WCD\left(S,S^{\prime }\right)=\sum_{n=1}^{L}w\left(n\right){\left(c_{n}-c_{n}^{\prime }\right)}^{2}, \end{array}$$ (9)

where *w*(*n*) is a lifter function and given as

$$\begin{array}{c} w\left(n\right)=\left\{\begin{array}{cc} 1+h\sin\left(\frac{n\pi }{L}\right) & :\;n=1,\ldots ,L\\ 0 & :\;n\leq 0,n>L, \end{array}\right. \end{array}$$ (10)

in which *h* is usually chosen as *L*/2, *L* is the truncated term and taken as *p* − 1 in this study, where *p* is the LPC number of poles, and the cepstral coefficients *c*_*n*_ is derived with the following recursion [27]:

$$\begin{array}{c} c_{n}=-a_{n}-\frac{1}{n}\;\sum_{k=1}^{n-1}k\;c_{n}\;a_{n-k},\;n>0, \end{array}$$ (11)

where *a*_*n*_, *n* = 1, …, *p*, are the LPC coefficients, *a*_0_ = 1, *a*_*k*_ = 1 for *k* > *p*, *c*_0_ = log *σ* ^2^, and *c*_−*n*_ = *c*_*n*_.

### Phylogenetic tree reconstruction

Molecular biology suggests that if genomes change slowly by the gradual accumulation of mutations, then the amount of difference in a nucleotide sequence between a pair of genomes should indicate how recently those two genomes shared a common ancestor [31]. In other words, it is expected that the dissimilarity of two genomes that diverged in the recent past would be less than a pair of genomes whose common ancestor is more ancient. Based on this hypothesis of evolution, molecular phylogenetics aims to infer the evolutionary relationships between three or more genomes by comparing their DNA sequences for classifying molecular data. Plotting a phylogenetic tree is helpful because one can easily visualize the evolutionary relationships between species. The notion of phylogenetic tree reconstruction has been applied to partition MRI white-matter-lesion patterns into similar groups, which can be helpful for studying age-related diseases [32].

Phylogenetic tree reconstruction can be done using a number of different tree-building models. A popular choice is the use of the dissimilarity/similarity matrix based approach, such as the unweighted pair-group method using arithmetic averaging (UPGMA) [33] for linking the tree nodes. UPGMA, which is a hierarchical cluster analysis, generates nested hard clusters in dataset **X** by merging the two clusters at each step based on the minimization of a dissimilarity measure. The UPGMA algorithm mathematically works as follows [34]:

1. Given **X** ∈ 𝓡^*q*^, *n* = ∣**X**∣, **U**_*n*_ = [*δ*_*ij*_]_*n*×*n*_, where *δ*_*ij*_ is the Kronecker delta: 0 if *i* ≠ *j* and 1 if *i* = *j* (each **x**_*k*_ ∈ **X** is a singleton cluster at the number of clusters *c* = *n*, **V**(*n*) = 0, where **V**(*n*) is the *c*-partition of **X**).
2. At step *k*, *k* = 1, …, *n* − 1, *c* = *n* − *k* + 1, using **U**_*c*_ to directly solve the measure of hard-cluster similarity (hard clustering means that each data point is a member of one and only one cluster) by minimizing the following function to identify the minimum distance as the similarity between any two data points in **X**:
  $$\begin{array}{c} \underset{j,k}{\text{minimize}}\;\left[J\left(u_{j},u_{k}\right)=\frac{\sum_{i=t+1}^{n}\sum_{t=1}^{n-1}\;u_{ji}\;u_{kt}\;d\left(x_{i},x_{t}\right)}{\left(\sum_{i=1}^{n}u_{ji}\right)\left(\sum_{t=1}^{n}u_{kt}\right)}\right], \end{array}$$ (12)
  where **u**_*j*_, **u**_*k*_ denote the *j*-th and *k*-th rows of **U**_*c*_, *u*_*ji*_ ∈ [0, 1], and *d*: **X** × **X** → 𝓡^+^ is any measure of dissimilarity on **X**, and *d* was used as a spectral-distortion measure in this study.
3. Let (**u**_*r*_, **u**_*s*_)_*c*_ solve Eq. (12). Merge *u*_*r*_ and *u*_*s*_, thus constructing from **U**_*c*_ the updated partition **U**_*c*−1_, record **V**(*c* − 1) = **J**[(**u**_*r*_, **u**_*s*_)_*c*_].
4. If *k* < *n* − 1, go to Step 2; if *k* = *n* − 1, *c* = 2. Merge the two remaining clusters, set **U**_1_ = [1, …, 1], compute **V**(*c* − 1) = **J**(**u**_1_, **u**_2_), and stop.

## Results and Discussion

**Fig 1:**
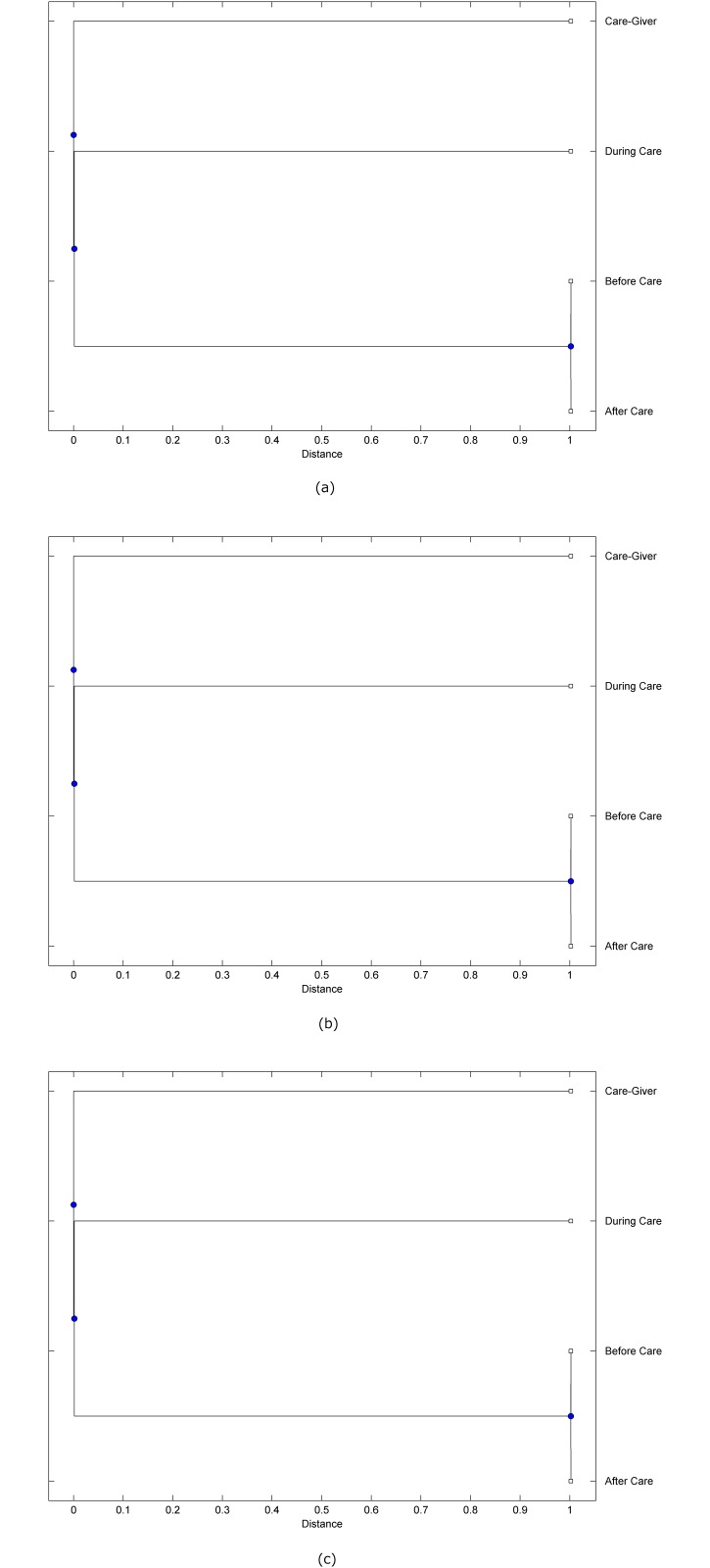
Assessment of synchronized cognitive stimulation communication between care-giver and Participant #1, where PPG data before and after care session are used as control variables. Dissimilarities of PPG data between care-giver and participant were determined by spectral-distortion measures: Itakura distortion (a), log spectral distortion (b), and weighted cepstral distortion (c). Matrices of dissimilarity are used to construct trees of relationships between PPG data of care-giver and participant before, during and after synchronized communication.

**Fig 2:**
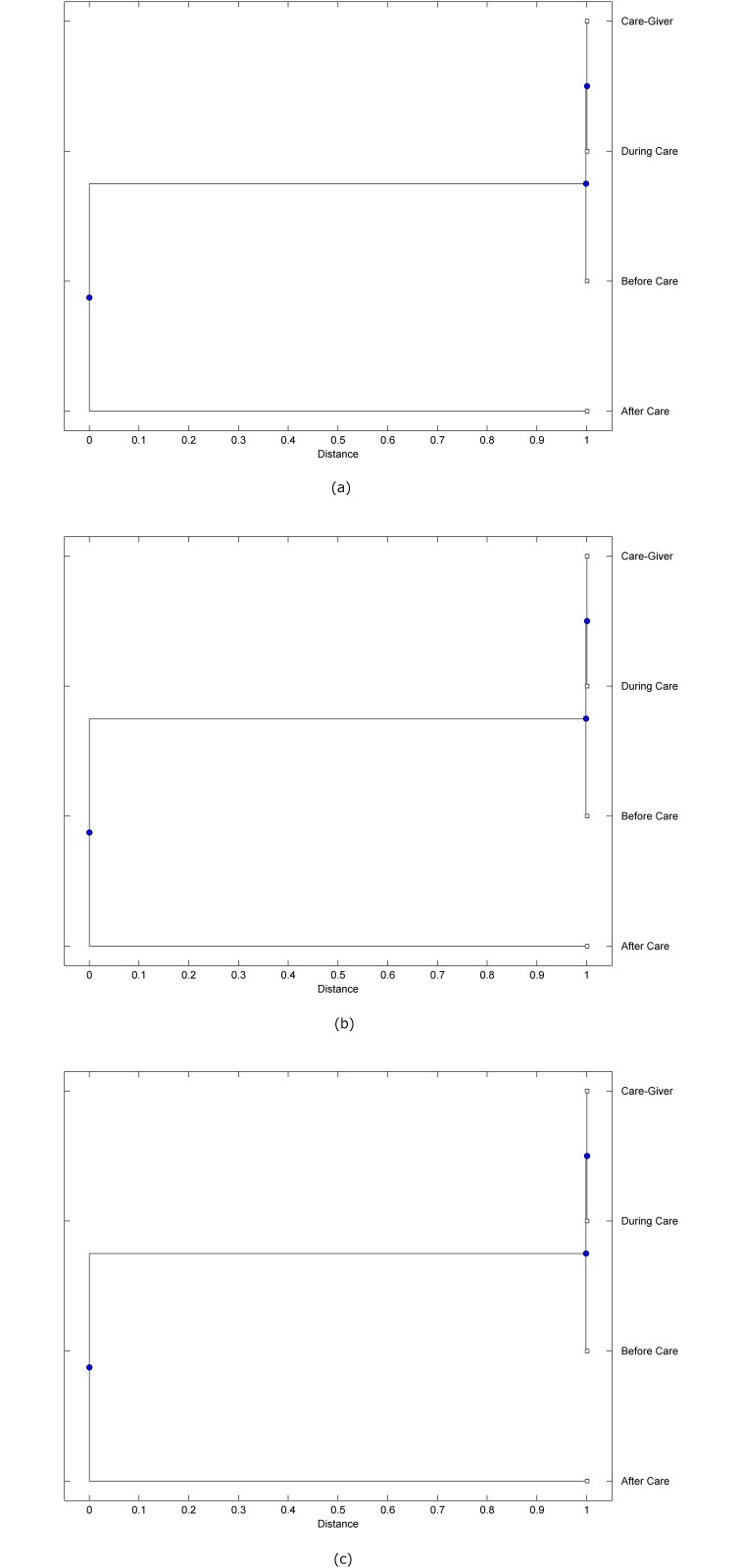
Assessment of synchronized cognitive stimulation communication between care-giver and Participant #2, where PPG data before and after care session are used as control variables. Dissimilarities of PPG data between care-giver and participant were determined by spectral-distortion measures: Itakura distortion (a), log spectral distortion (b), and weighted cepstral distortion (c). Matrices of dissimilarity are used to construct trees of relationships between PPG data of care-giver and participant before, during and after synchronized communication.

**Fig 3:**
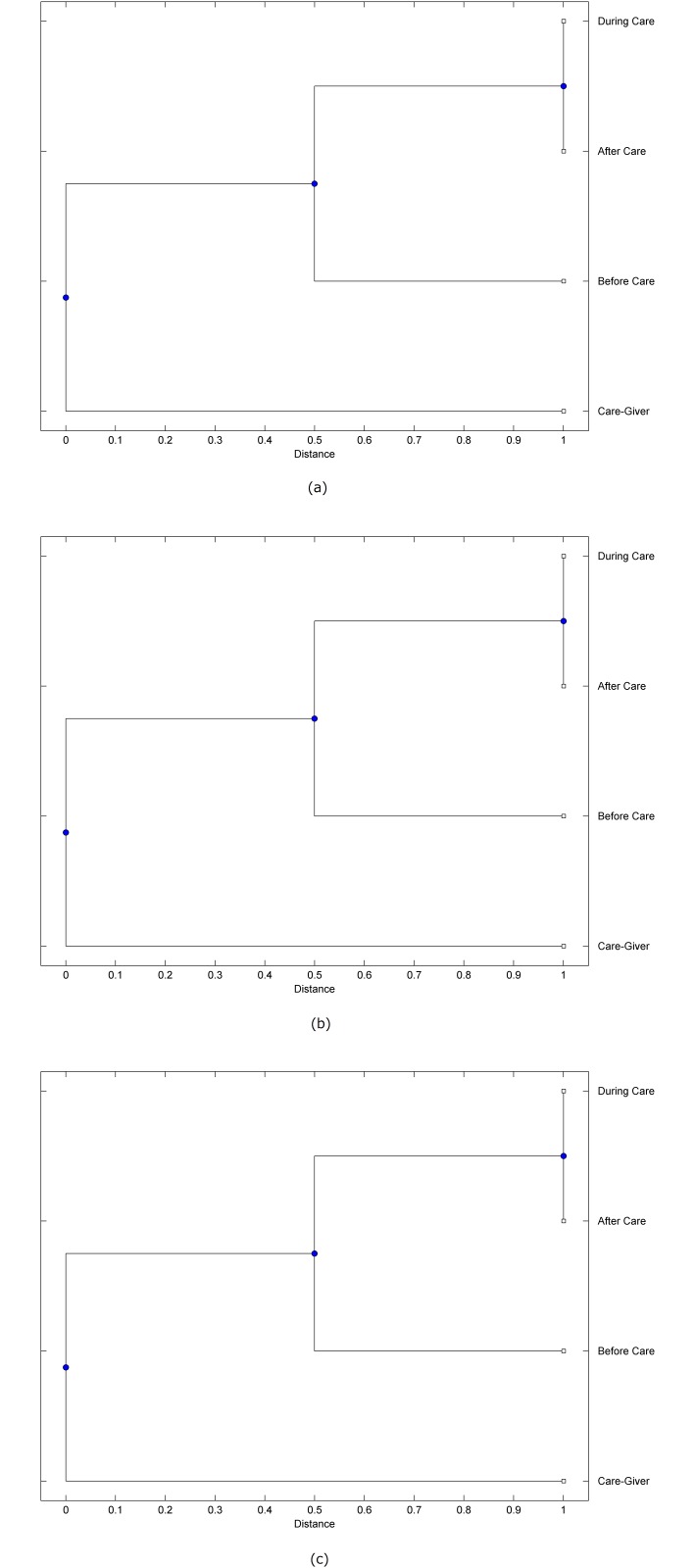
Assessment of synchronized cognitive stimulation communication between care-giver and Participant #3, where PPG data before and after care session are used as control variables. Dissimilarities of PPG data between care-giver and participant were determined by spectral-distortion measures: Itakura distortion (a), log spectral distortion (b), and weighted cepstral distortion (c). Matrices of dissimilarity are used to construct trees of relationships between PPG data of care-giver and participant before, during and after synchronized communication.

**Fig 4:**
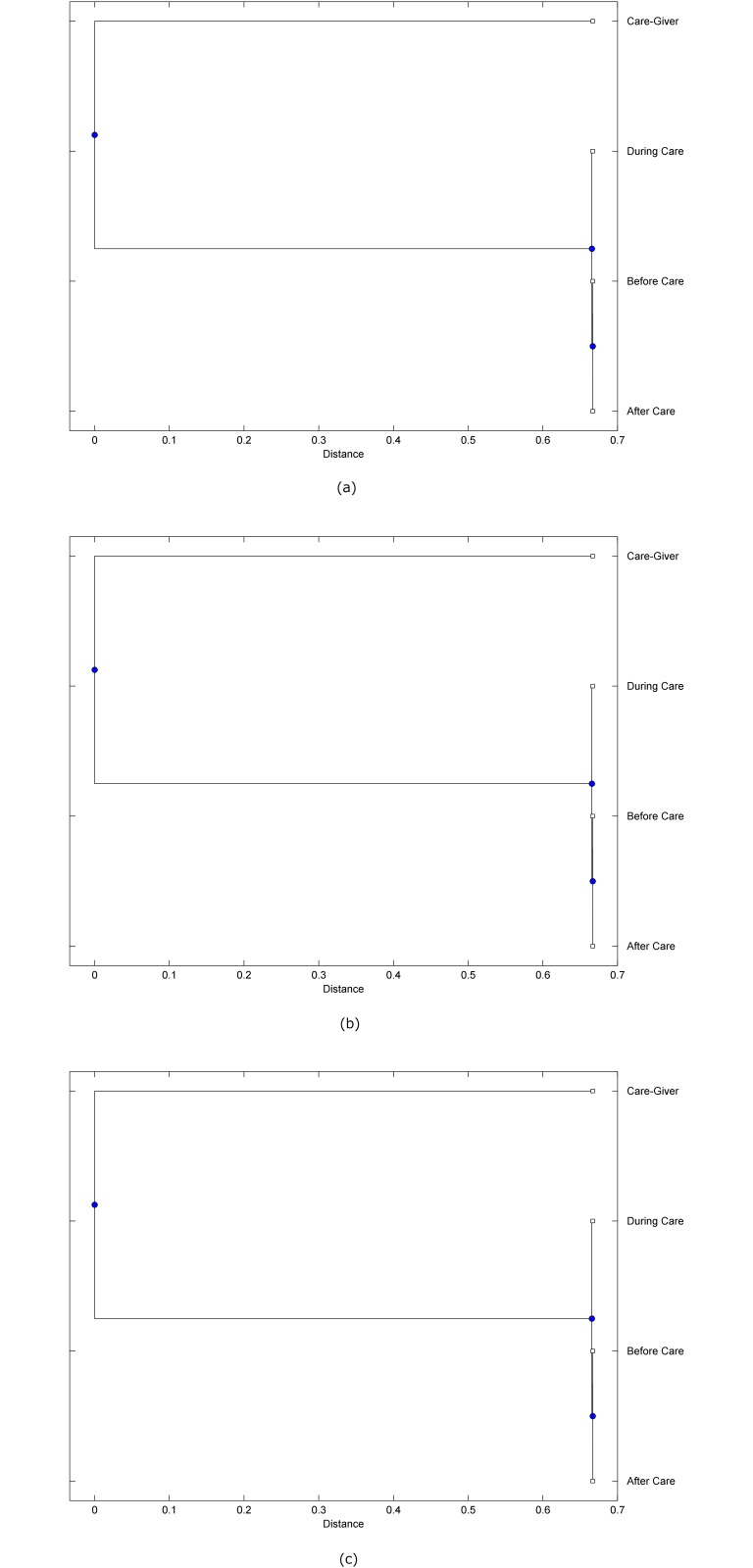
Assessment of synchronized cognitive stimulation communication between care-giver and Participant #4, where PPG data before and after care session are used as control variables. Dissimilarities of PPG data between care-giver and participant were determined by spectral-distortion measures: Itakura distortion (a), log spectral distortion (b), and weighted cepstral distortion (c). Matrices of dissimilarity are used to construct trees of relationships between PPG data of care-giver and participant before, during and after synchronized communication.

**Fig 5:**
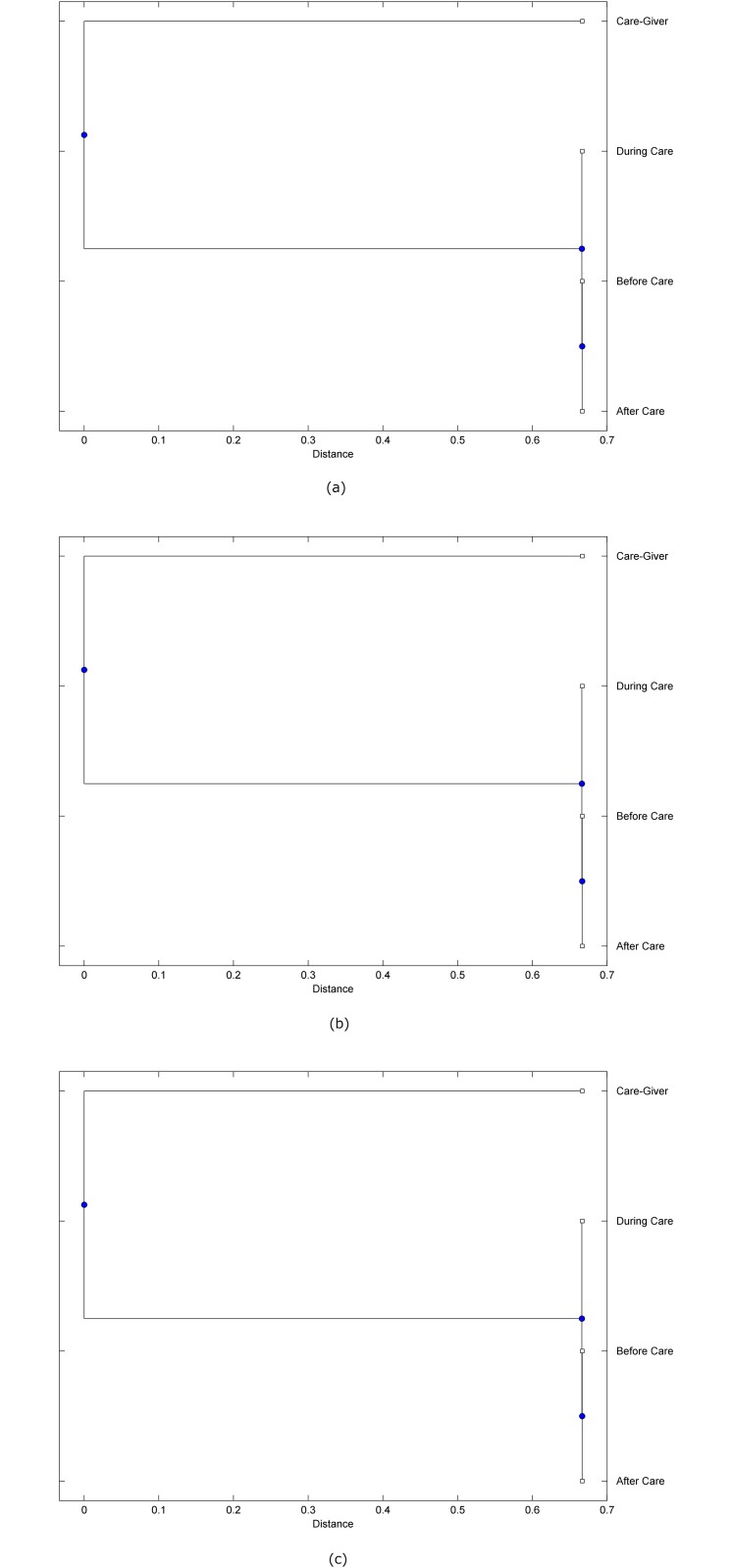
Assessment of synchronized cognitive stimulation communication between care-giver and Participant #5, where PPG data before and after care session are used as control variables. Dissimilarities of PPG data between care-giver and participant were determined by spectral-distortion measures: Itakura distortion (a), log spectral distortion (b), and weighted cepstral distortion (c). Matrices of dissimilarity are used to construct trees of relationships between PPG data of care-giver and participant before, during and after synchronized communication.

**Fig 6:**
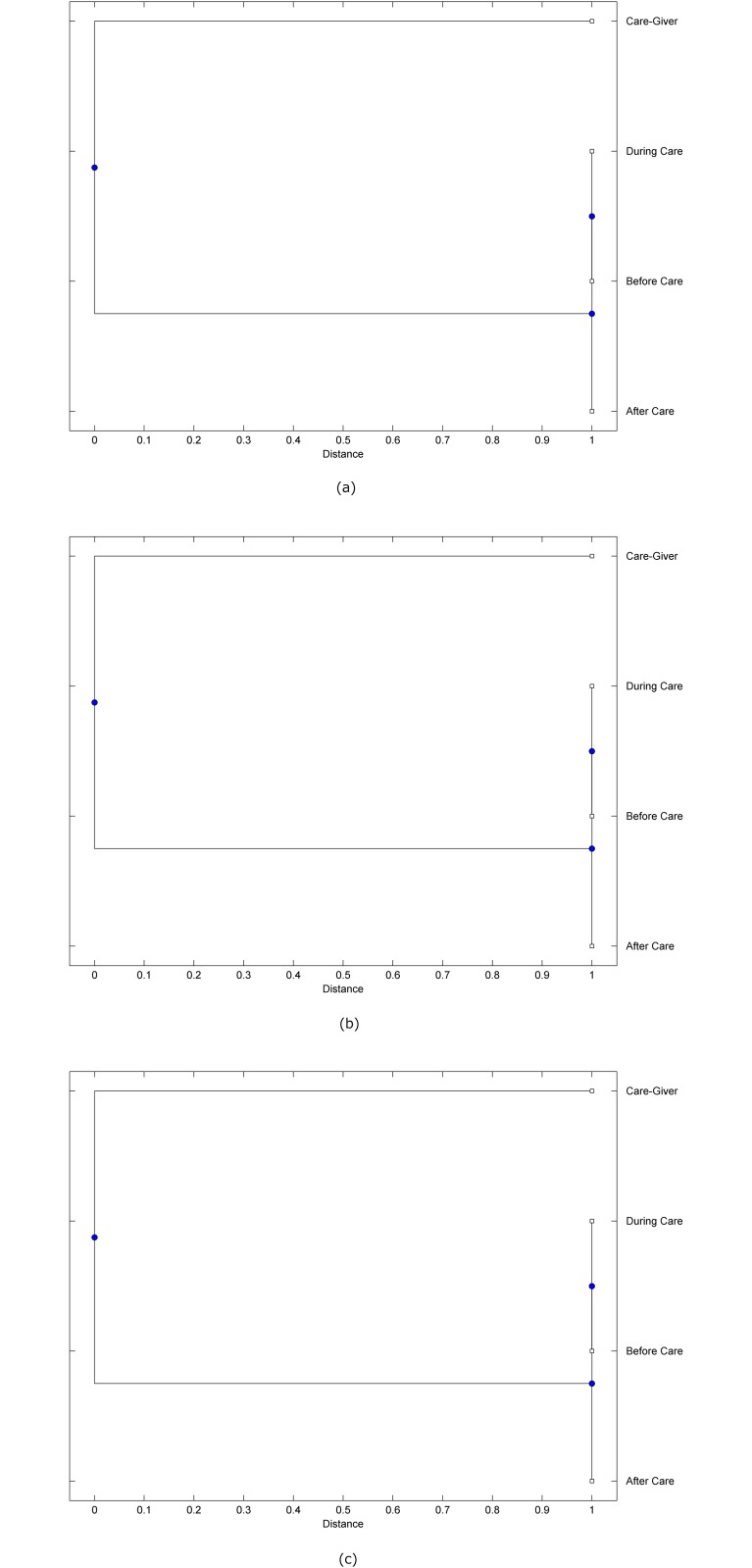
Assessment of synchronized cognitive stimulation communication between care-giver and Participant #6, where PPG data before and after care session are used as control variables. Dissimilarities of PPG data between care-giver and participant were determined by spectral-distortion measures: Itakura distortion (a), log spectral distortion (b), and weighted cepstral distortion (c). Matrices of dissimilarity are used to construct trees of relationships between PPG data of care-giver and participant before, during and after synchronized communication.

**Fig 7:**
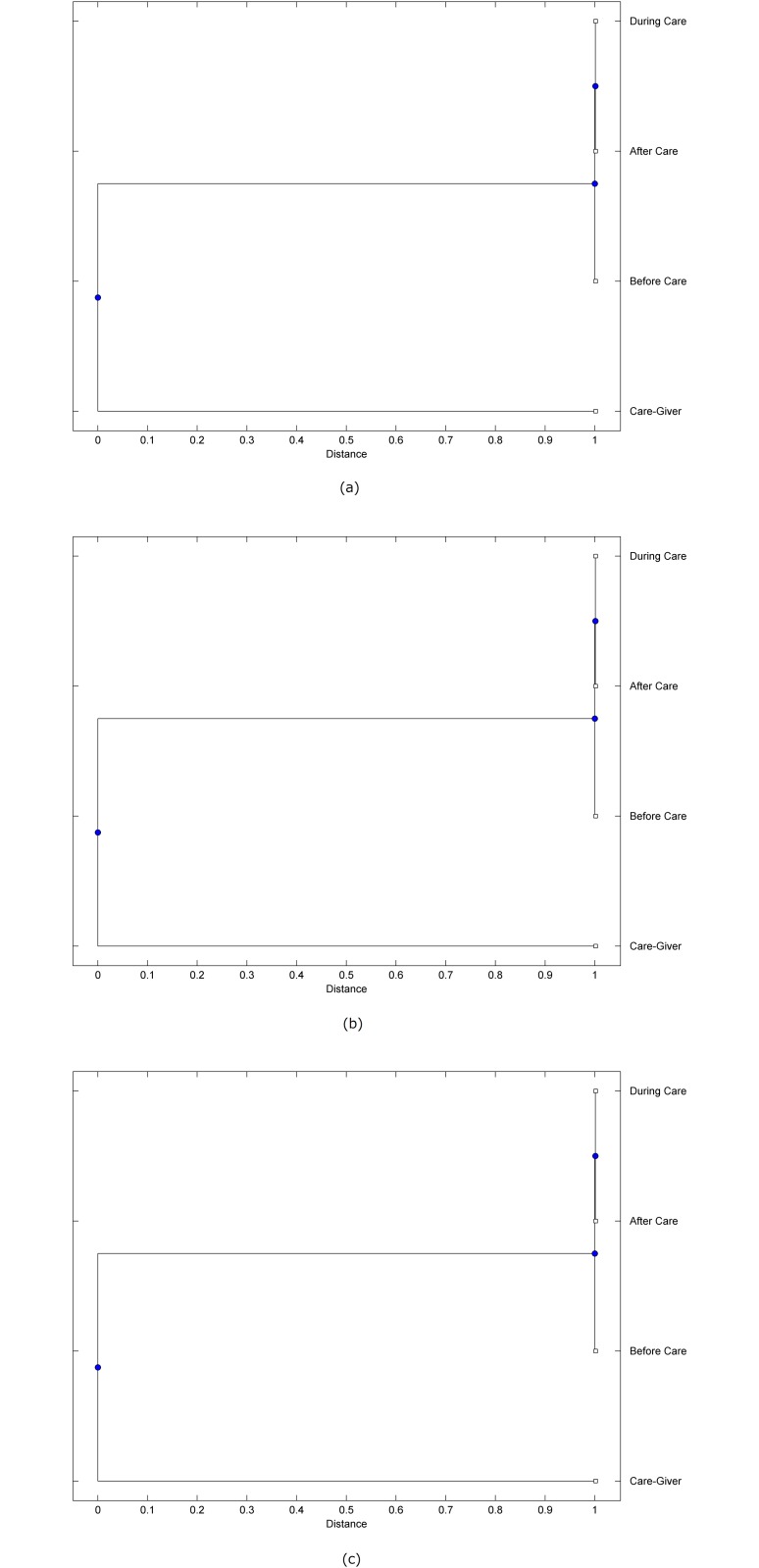
Assessment of synchronized cognitive stimulation communication between care-giver and Participant #7, where PPG data before and after care session are used as control variables. Dissimilarities of PPG data between care-giver and participant were determined by spectral-distortion measures: Itakura distortion (a), log spectral distortion (b), and weighted cepstral distortion (c). Matrices of dissimilarity are used to construct trees of relationships between PPG data of care-giver and participant before, during and after synchronized communication.

**Fig 8:**
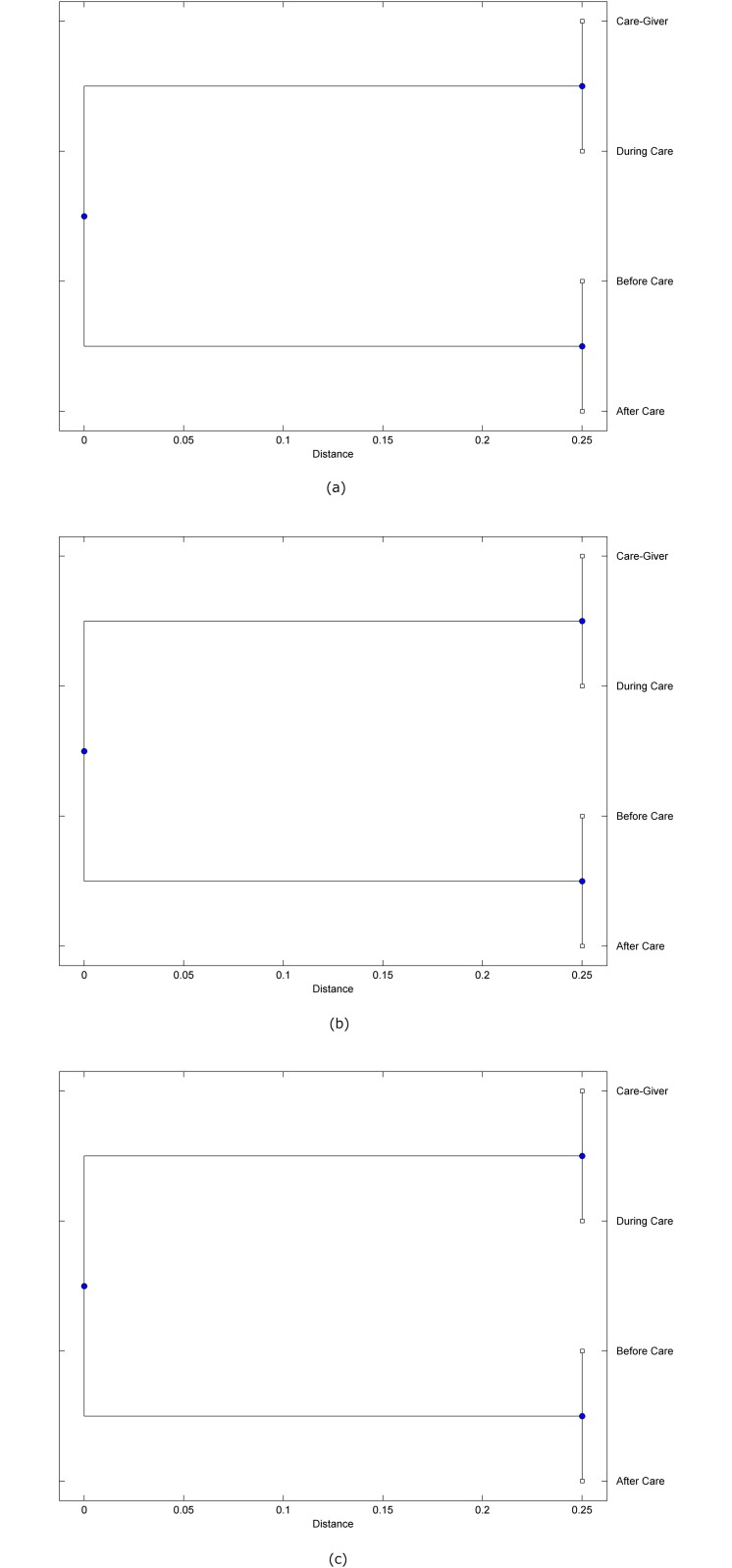
Assessment of synchronized cognitive stimulation communication between care-giver and Participant #8, where PPG data before and after care session are used as control variables. Dissimilarities of PPG data between care-giver and participant were determined by spectral-distortion measures: Itakura distortion (a), log spectral distortion (b), and weighted cepstral distortion (c). Matrices of dissimilarity are used to construct trees of relationships between PPG data of care-giver and participant before, during and after synchronized communication.

**Fig 9:**
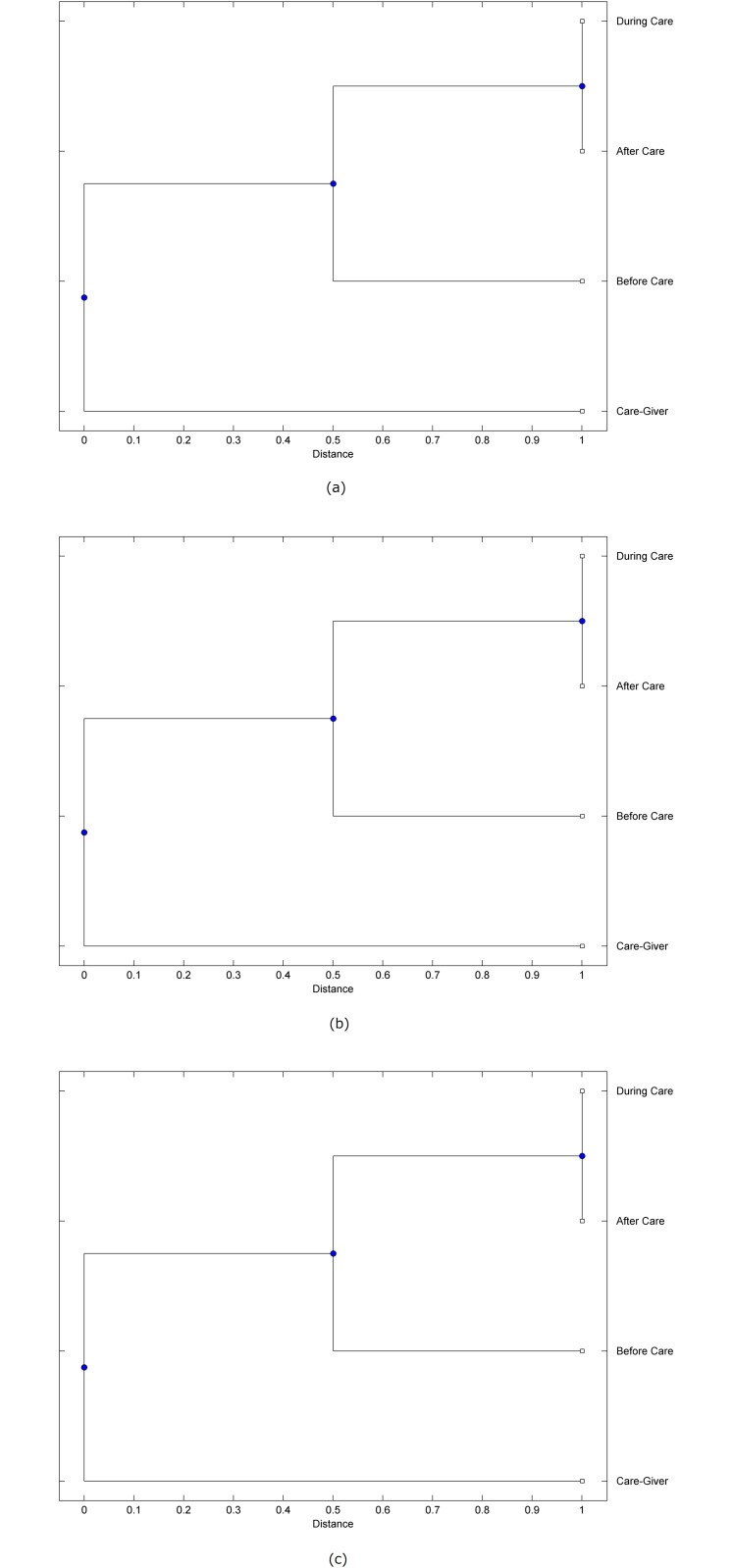
Assessment of synchronized cognitive stimulation communication between care-giver and Participant #9, where PPG data before and after care session are used as control variables. Dissimilarities of PPG data between care-giver and participant were determined by spectral-distortion measures: Itakura distortion (a), log spectral distortion (b), and weighted cepstral distortion (c). Matrices of dissimilarity are used to construct trees of relationships between PPG data of care-giver and participant before, during and after synchronized communication.

**Fig 10:**
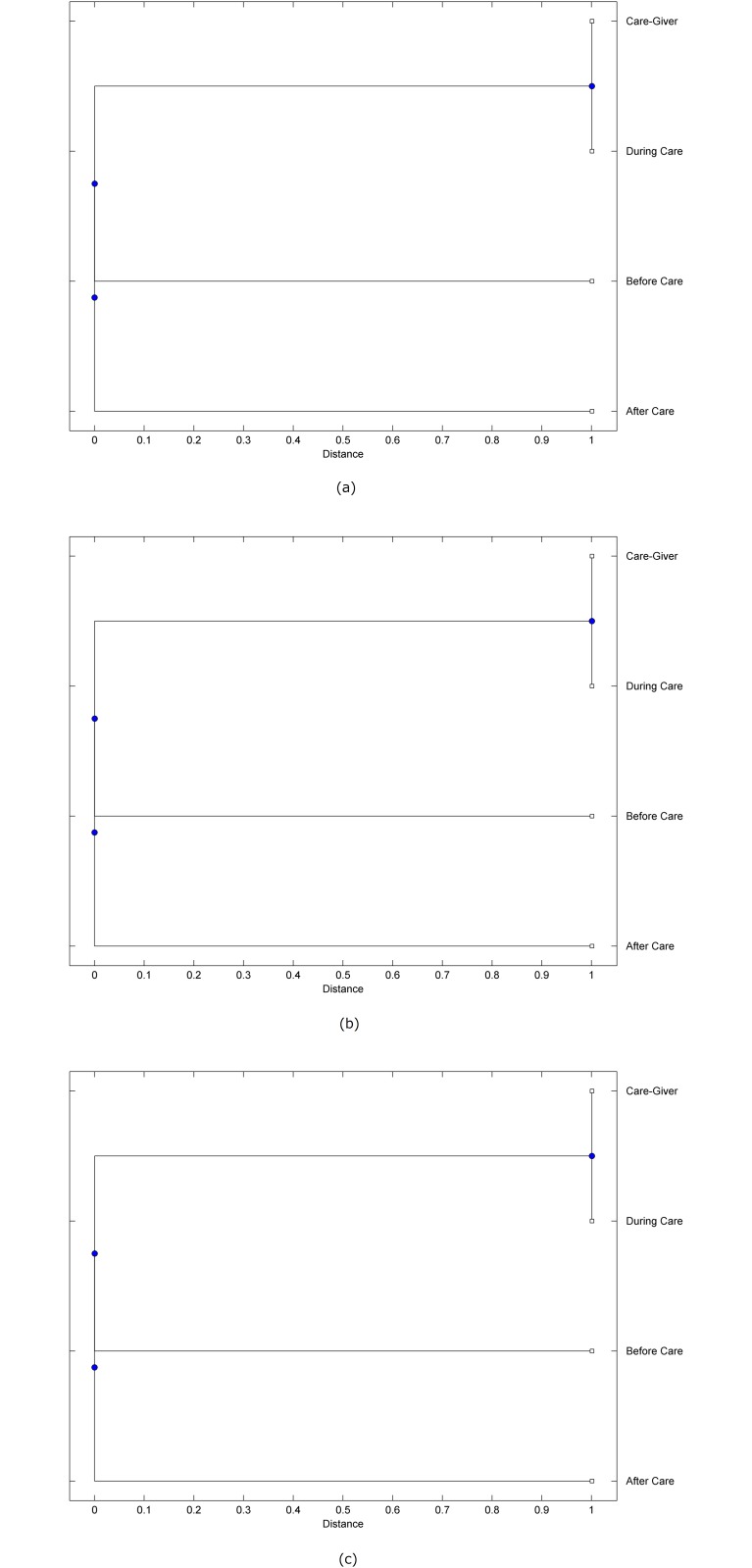
Assessment of synchronized cognitive stimulation communication between care-giver and Participant #10, where PPG data before and after care session are used as control variables. Dissimilarities of PPG data between care-giver and participant were determined by spectral-distortion measures: Itakura distortion (a), log spectral distortion (b), and weighted cepstral distortion (c). Matrices of dissimilarity are used to construct trees of relationships between PPG data of care-giver and participant before, during and after synchronized communication.

**Fig 11:**
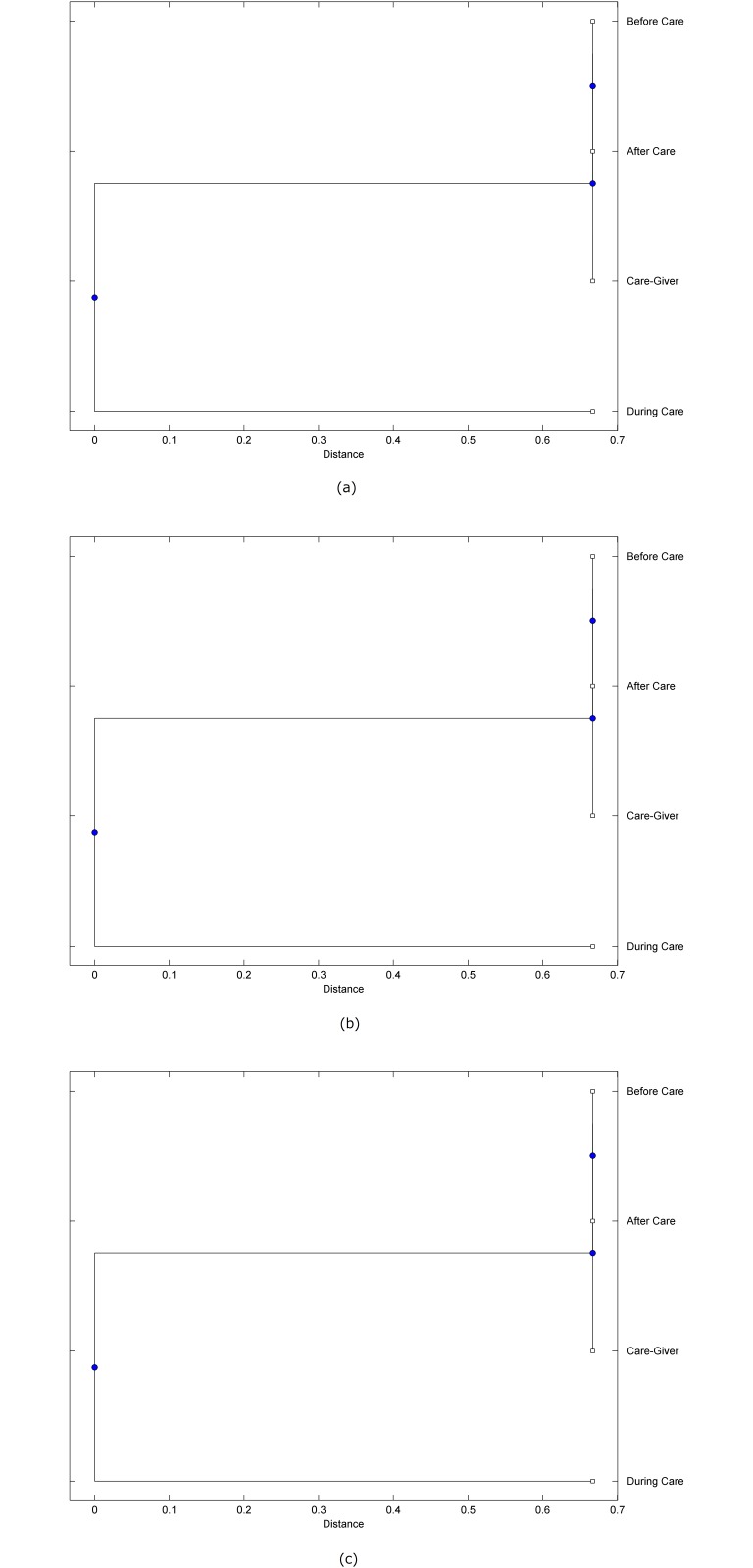
Assessment of synchronized cognitive stimulation communication between care-giver and Participant #11, where PPG data before and after care session are used as control variables. Dissimilarities of PPG data between care-giver and participant were determined by spectral-distortion measures: Itakura distortion (a), log spectral distortion (b), and weighted cepstral distortion (c). Matrices of dissimilarity are used to construct trees of relationships between PPG data of care-giver and participant before, during and after synchronized communication.

**Fig 12:**
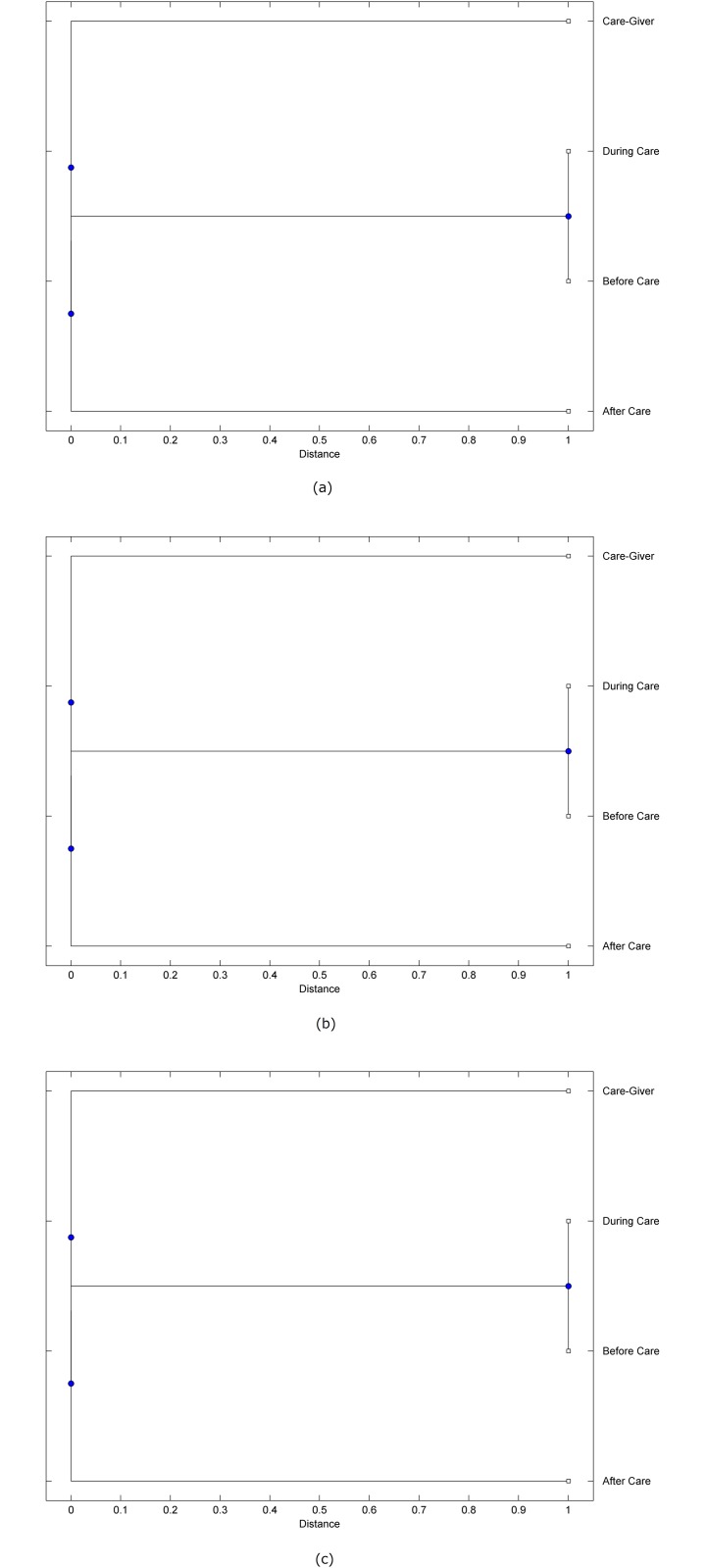
Assessment of synchronized cognitive stimulation communication between care-giver and Participant #12, where PPG data before and after care session are used as control variables. Dissimilarities of PPG data between care-giver and participant were determined by spectral-distortion measures: Itakura distortion (a), log spectral distortion (b), and weighted cepstral distortion (c). Matrices of dissimilarity are used to construct trees of relationships between PPG data of care-giver and participant before, during and after synchronized communication.

**Fig 13:**
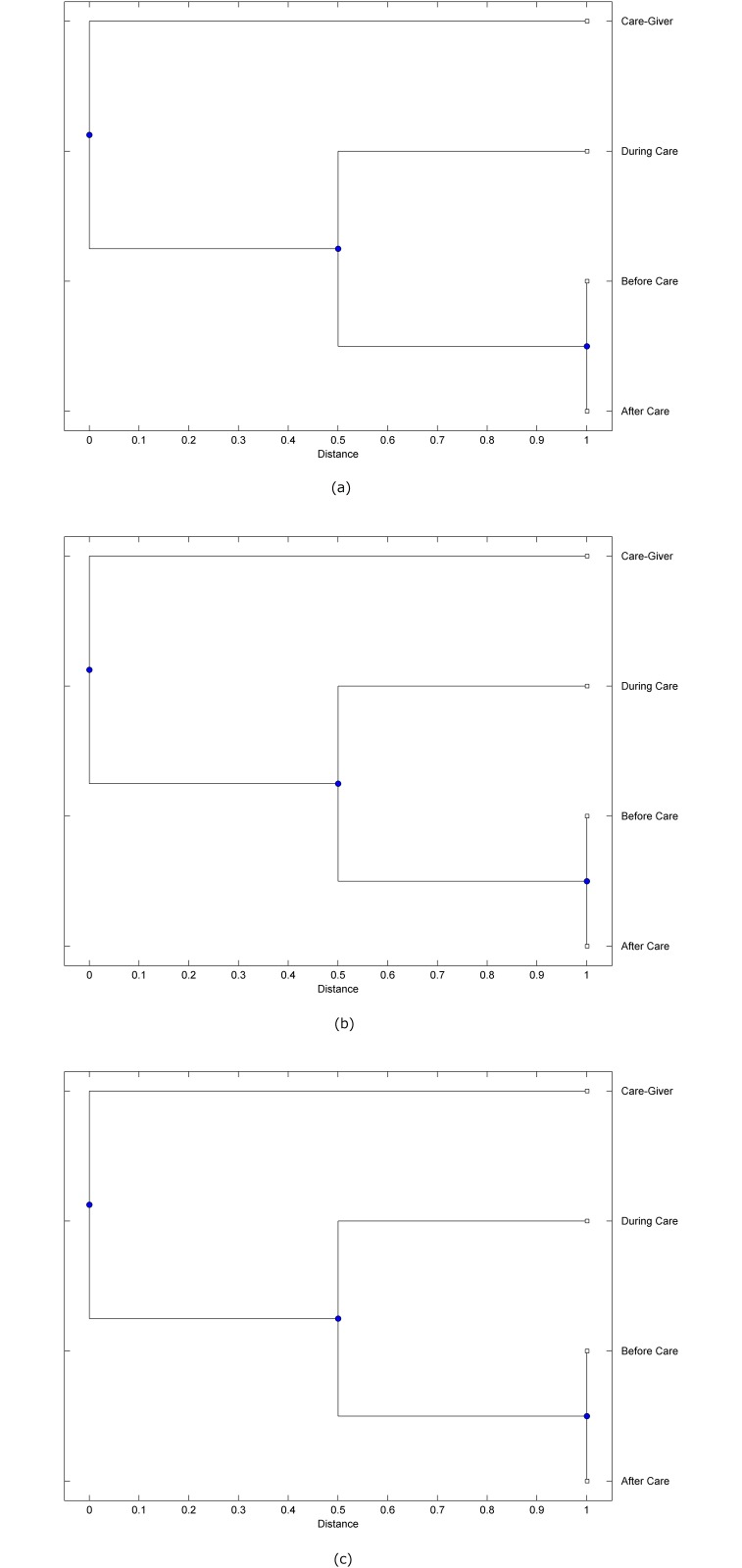
Assessment of synchronized cognitive stimulation communication between care-giver and Participant #13, where PPG data before and after care session are used as control variables. Dissimilarities of PPG data between care-giver and participant were determined by spectral-distortion measures: Itakura distortion (a), log spectral distortion (b), and weighted cepstral distortion (c). Matrices of dissimilarity are used to construct trees of relationships between PPG data of care-giver and participant before, during and after synchronized communication.

**Fig 14:**
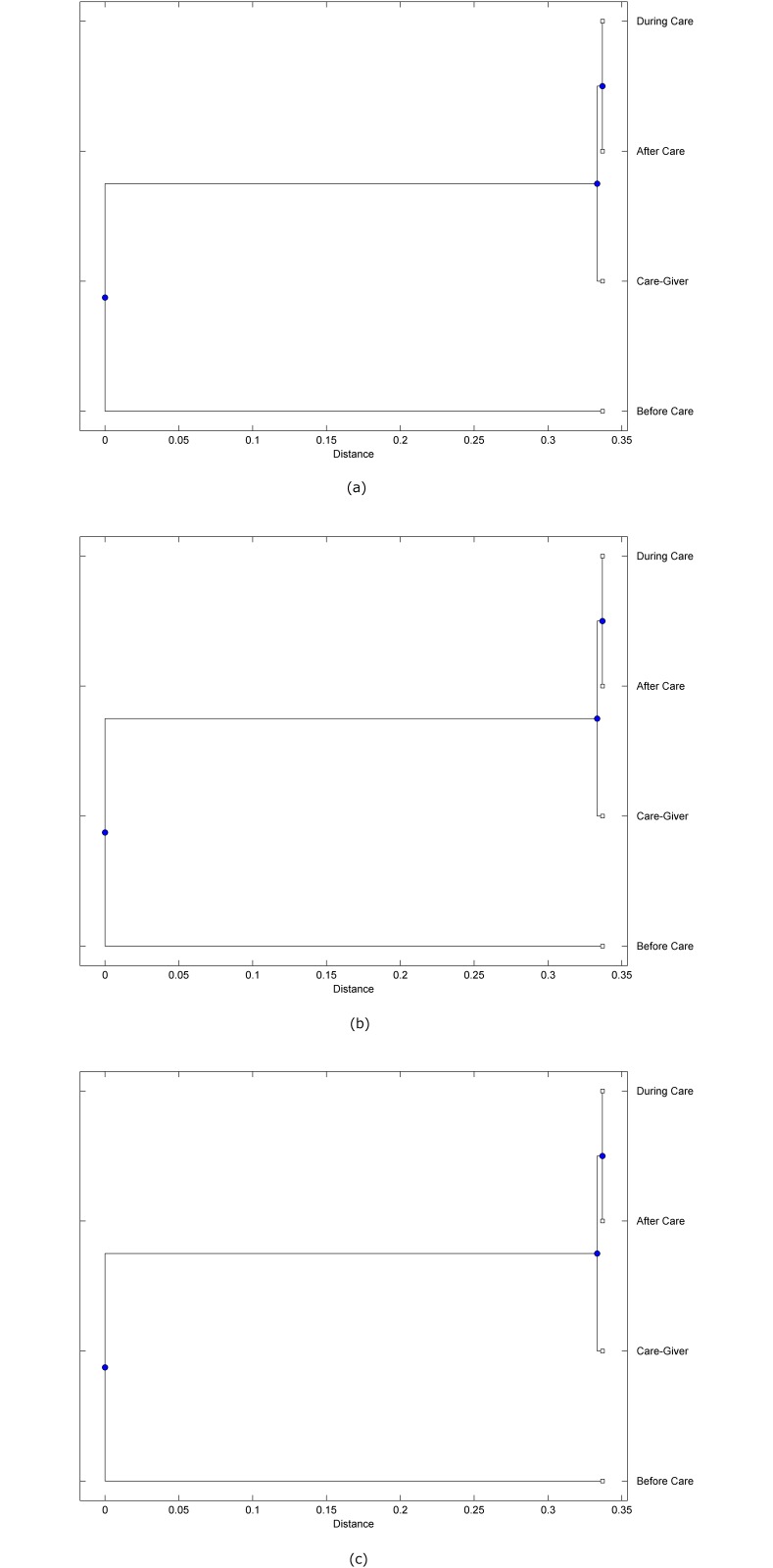
Assessment of synchronized cognitive stimulation communication between care-giver and Participant #14, where PPG data before and after care session are used as control variables. Dissimilarities of PPG data between care-giver and participant were determined by spectral-distortion measures: Itakura distortion (a), log spectral distortion (b), and weighted cepstral distortion (c). Matrices of dissimilarity are used to construct trees of relationships between PPG data of care-giver and participant before, during and after synchronized communication.

**Fig 15:**
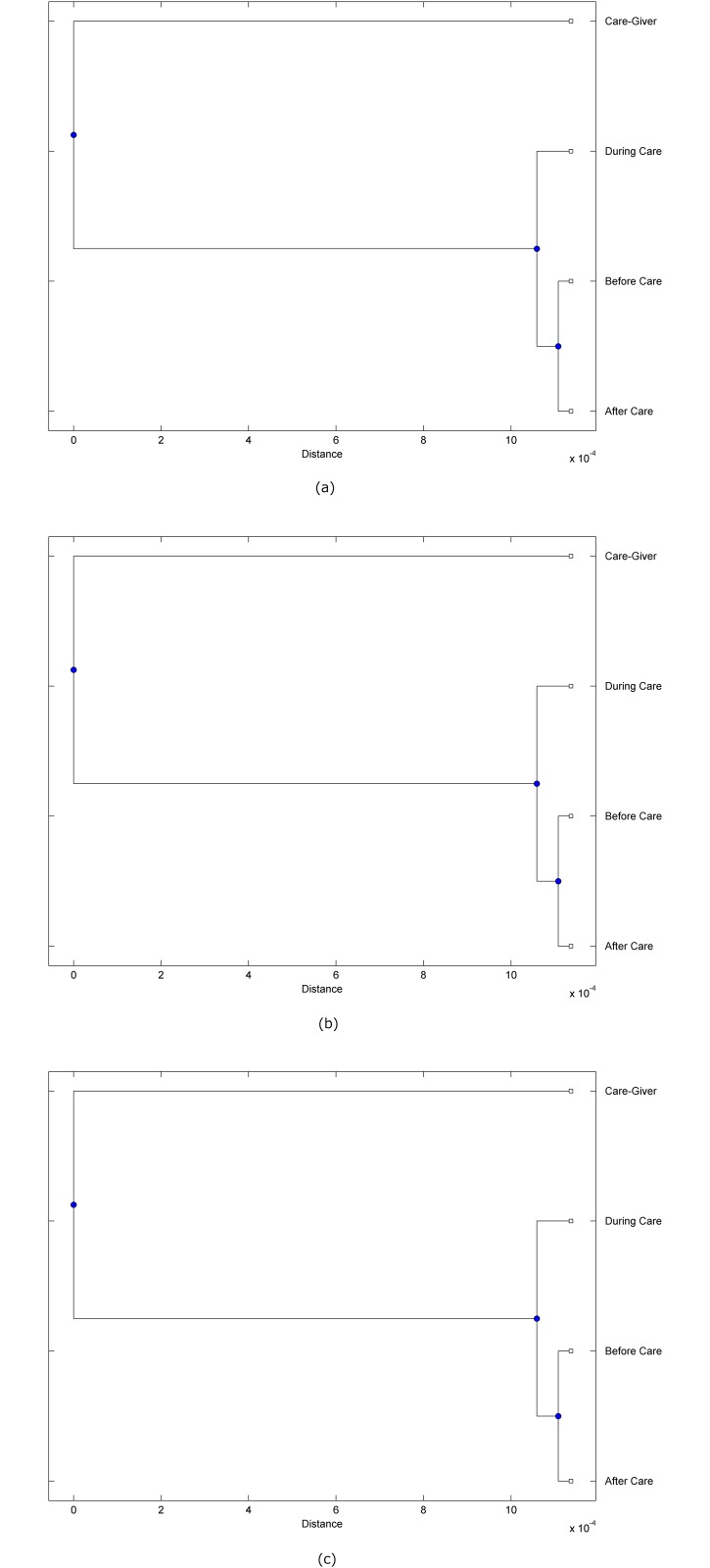
Assessment of synchronized cognitive stimulation communication between care-giver and Participant #15, where PPG data before and after care session are used as control variables. Dissimilarities of PPG data between care-giver and participant were determined by spectral-distortion measures: Itakura distortion (a), log spectral distortion (b), and weighted cepstral distortion (c). Matrices of dissimilarity are used to construct trees of relationships between PPG data of care-giver and participant before, during and after synchronized communication.

**Fig 16:**
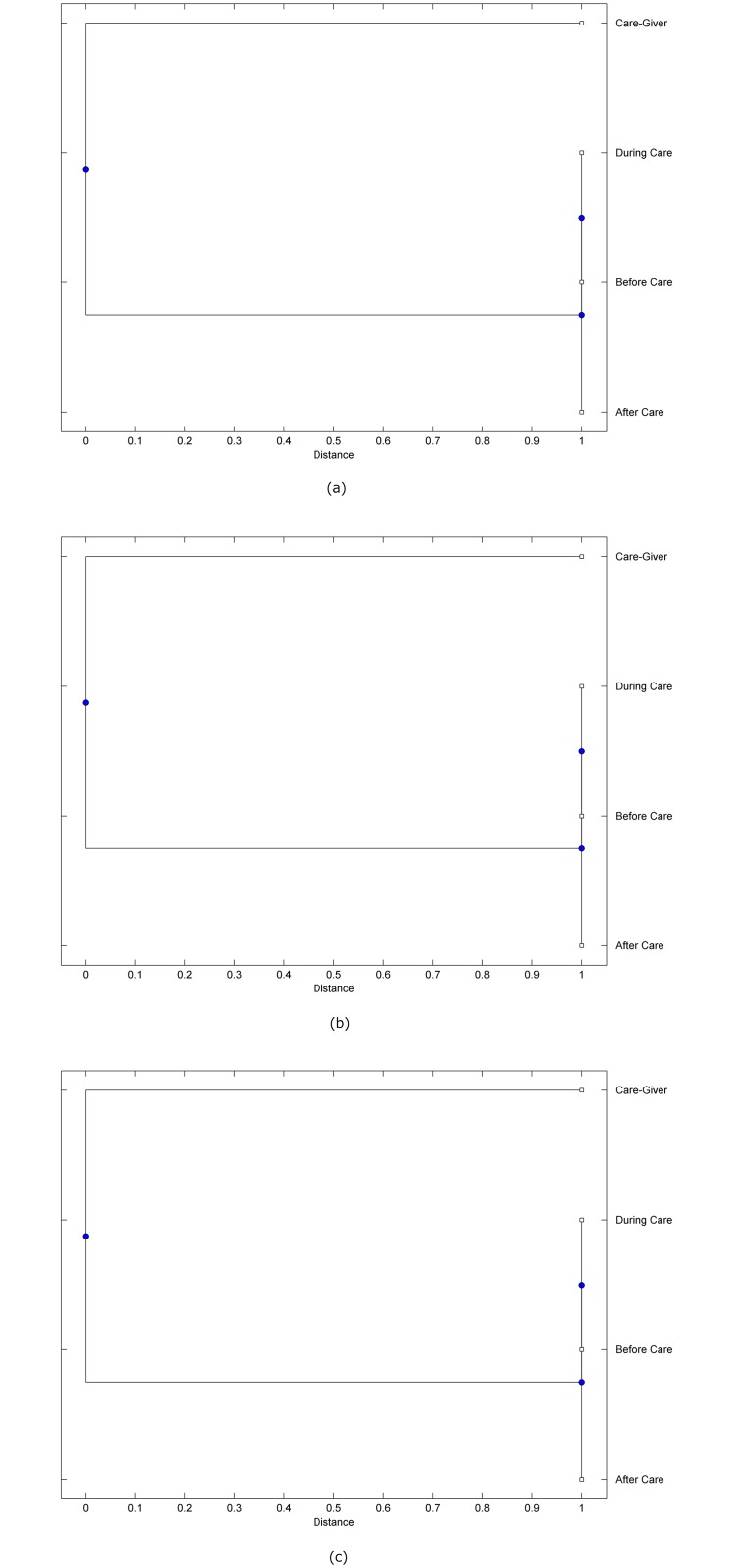
Assessment of synchronized cognitive stimulation communication between care-giver and Participant #16, where PPG data before and after care session are used as control variables. Dissimilarities of PPG data between care-giver and participant were determined by spectral-distortion measures: Itakura distortion (a), log spectral distortion (b), and weighted cepstral distortion (c). Matrices of dissimilarity are used to construct trees of relationships between PPG data of care-giver and participant before, during and after synchronized communication.

**Fig 17:**
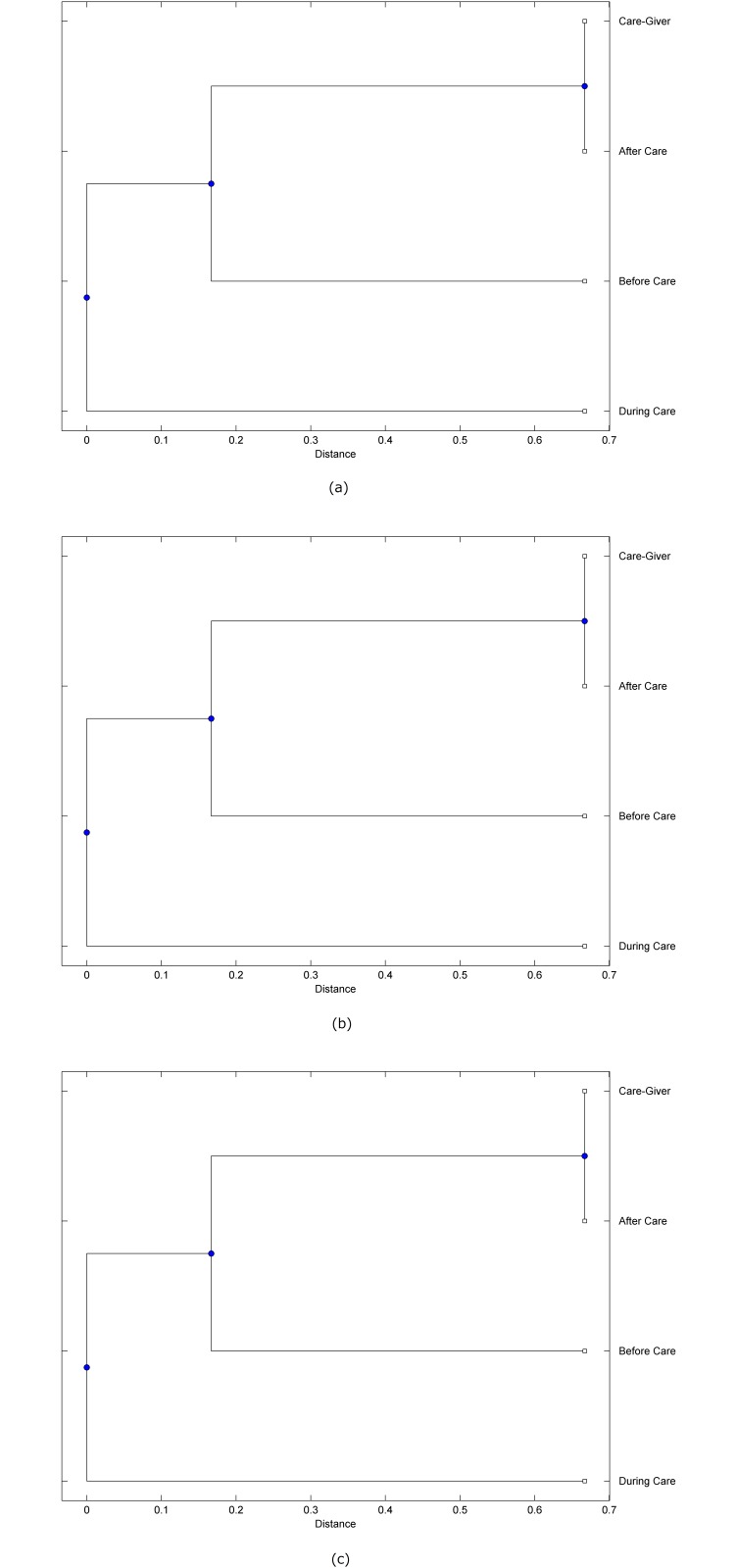
Assessment of synchronized cognitive stimulation communication between care-giver and Participant #17, where PPG data before and after care session are used as control variables. Dissimilarities of PPG data between care-giver and participant were determined by spectral-distortion measures: Itakura distortion (a), log spectral distortion (b), and weighted cepstral distortion (c). Matrices of dissimilarity are used to construct trees of relationships between PPG data of care-giver and participant before, during and after synchronized communication.

**Fig 18:**
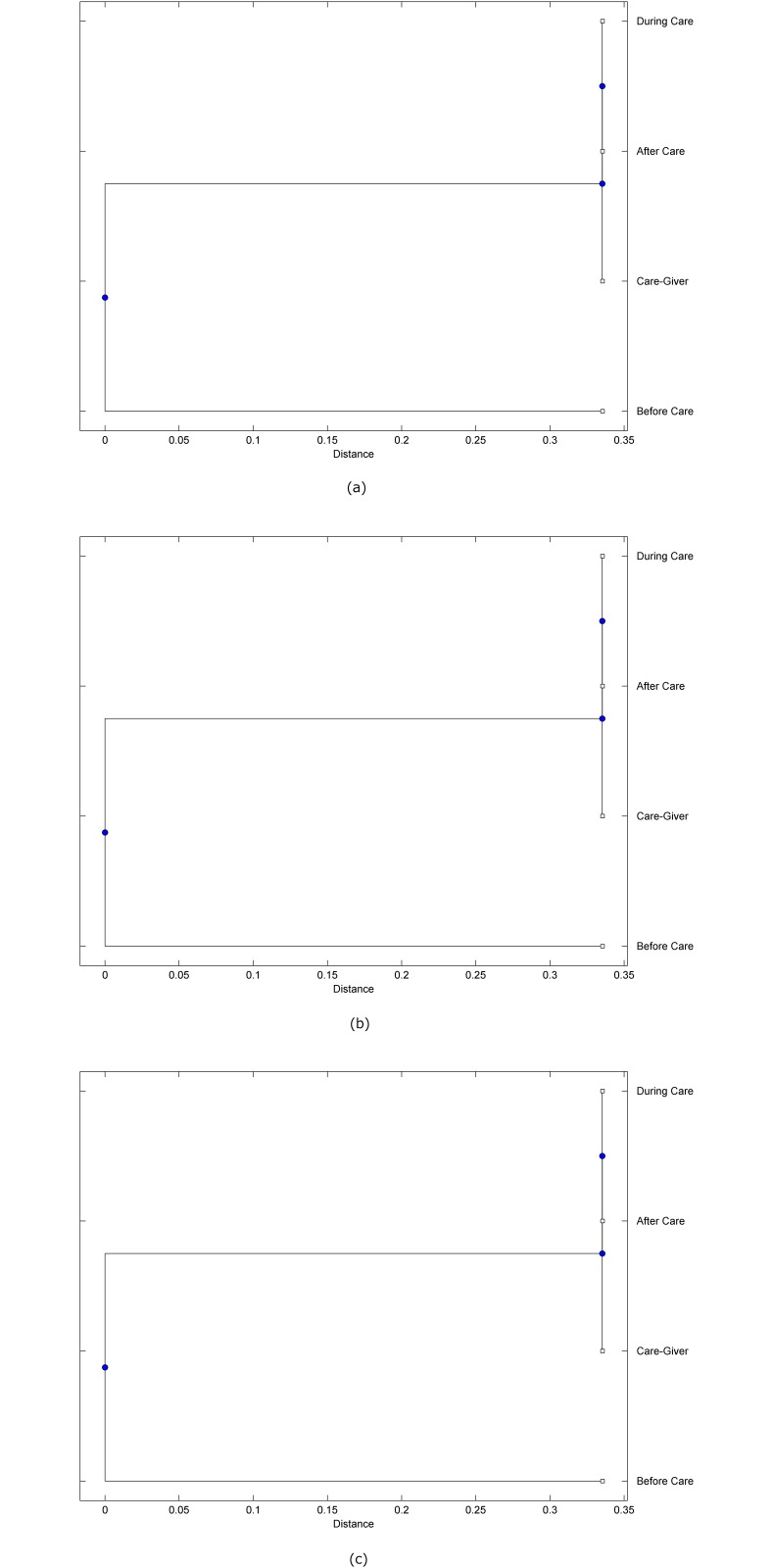
Assessment of synchronized cognitive stimulation communication between care-giver and Participant #18, where PPG data before and after care session are used as control variables. Dissimilarities of PPG data between care-giver and participant were determined by spectral-distortion measures: Itakura distortion (a), log spectral distortion (b), and weighted cepstral distortion (c). Matrices of dissimilarity are used to construct trees of relationships between PPG data of care-giver and participant before, during and after synchronized communication.

The preprocessed PPG data of the care-giver and 18 selected participants were used to calculate the three spectral-distortion measures (ID, LSD, and WCD) between the care-giver and each of the participants before, during and after the therapeutic session. The 12th-order LPC model (*p* = 12) was used to calculate the LPC coefficients. The dissimilarity matrices of the PPG data between the care-giver and the participants obtained from the three distortion measures were then used to construct the “phylogenetic” trees with the UPGMA algorithm. Figs. 1–18 show the trees of the PPG data of 18 participants and the care-giver, in which the terms *Care-giver*, *Before care*, *During care*, and *After care* in the tree nodes denote the care-giver, the participated individual before, during, and after the therapeutic session, respectively. A phylogenetic tree is composed of branches (edges) and nodes. Branches connect nodes each of which is the point at which two (or more) branches diverge. In the case of molecular phylogeny, trees are built for assigning similar species into the same groups. A node is a clade or a monophyletic group. All members of a tree node are assumed to have inherited a set of unique common characters [35]. Thus, based on the inference of the phylogenetic tree reconstruction, the evidence of influence of the care-giver over a particular participant is when the PPG patterns of the care-giver and the participant during the session belong to the same node. The three distortion measures were applied to construct the trees and used as the consensus of evidence for validating the computerized assessment.

Among the selection of 18 participants, the influence of the care-giver over Participant #8 is supported by all three distortion measures (3 out of 3 = 100%), as shown in Fig. 8. Support of the care-giver’s influence is partial over Participant #1, as Fig. 1 shows that the spectral patterns of the care-giver and participant during care are in the same node using ID and WCD (2 out of 3 = 67%). The effectiveness of the care-giver over Participant #2 is only supported by the ID measure (1 out of 3 = 33% as shown in Fig. 2). Although the PPG patterns of the care-giver and Participant #7 (Fig. 7) are not located in the same node, the pattern of the care giver is closer to those of the participant’s during-care and after-care branches that connect the same node, this topology should be considered as an evidence of the influence of the care-giver over the participant. The influence of the care-giver over Participant #9 is clearly supported by the WCD measure, based on the topology of the tree shown in the bottom of Fig. 9, which also shows the support of the LSD measure (middle tree of Fig. 9). The influence of the care-giver over Participant #10 has the same consensus rate (67%) with Participant #9, but the former was supported by the ID and LSD measures (Fig. 10). The LSD measure gives evidence that the patterns of the care-giver and Participant #13 are best-matched among other patterns (middle tree in Fig. 13), while there is a lack of support from the results given by the other two distortion measures (top and bottom trees of Fig. 13). The pattern of support of the influence over Participant #14, which is given by the ID measure (top tree shown in Fig. 14, is similar to that of Participant #7, given by the LSD measure (middle tree shown in Fig. 7). No obvious support by any of the three distortion measures can be found in the trees of the care-giver and Participants #3 (Fig. 3), #4 (Fig. 4), #5 (Fig. 5), #6 (Fig. 6), #11 (Fig. 11), #12 (Fig. 12), and #15–#18 (Fig. 15–Fig. 18).

It should be pointed out that, for the comparison of DNA or protein sequences, a popular and simple test of phylogenetic accuracy is usually carried out by the bootstrap method [35, 36]. Bootstrap analysis essentially tests whether the whole dataset supports the tree by means of creating multiple pseudo-datasets that are randomly sampled with replacement. Individual phylogenetic trees are then reconstructed from each of the pseudo-datasets, which are finally used to find the consensus of support for the data grouping. In principle, the bootstrap procedure randomly selects samples with replacement from a dataset, given a condition that the number of elements in each bootstrap sample equals the number of elements in the original dataset. Thus, a particular data point from the original dataset has a chance to appear multiple times in a bootstrap sample. Unlike DNA or protein sequences, the PPG patterns in this study are represented with vectors of spectral coefficients, and the application of the bootstrap method for these type of data is not simply applicable. Therefore, several distortion measures of the PPG patterns are applied to validate the results obtained from the tree topologies. Table 1 shows the consensus of support (%) of the care-giver’s influence over the 18 participants during cognitive stimulation therapy, including each participant’s identity number (ID), age, gender, as well as the spectral-distortion method(s) supporting the evidence.

**Table 1:** Consensus of support of care-giver’s influence over 18 participants during one-on-one cognitive stimulation therapy.

Participant ID#	Age	Gender	Consensus (%)	Supporting method(s)
1	84	Male	67	ID, WCD
2	92	Male	33	ID, WCD
3	89	Female	0	None
4	94	Female	0	None
5	85	Female	0	None
6	86	Female	0	None
7	93	Female	33	LSD
8	85	Female	100	ID, LSD, WCD
9	82	Female	67	LSD, WCD
10	88	Female	67	ID, LSD
11	77	Female	0	None
12	82	Female	0	None
13	86	Female	33	LSD
14	84	Female	33	ID
15	75	Female	0	None
16	94	Female	0	None
17	83	Female	0	None
18	95	Female	0	None

## Conclusion

Cognitive stimulation therapy has been widely discussed in literature, in particular for people with dementia [37]–[41]. This study adopted the use of synchronized PPG measurement for assessing the effectiveness of the communication for cognitive stimulation therapy based on the computational models of spectral distortion and phylogenetic inference. Experimental results of this pilot study show its potential application as an assistive computer-technology tool for assessing the efficacy of cognitive therapy. Because the PPG measurements can be synchronized among the care-giver or therapist and multiple participants, the approach can also be applied for the assessment of the effectiveness of group therapy.

Furthermore, research findings have suggested that the increase in the sense of life quality of disabled elderly is an important psychological factor for alleviating care-givers’ burden in Japan [42], the use of this proposed methodology can be applied as a feasible and cost-effective analysis for quantifying the relationship between life quality and burden among care-givers.

Three computational models, which are known as spectral-distortion measures, were applied in this pilot study to obtain the consensus of the “phylogenetic” tree results. The inclusion of other potential methods for pattern matching of PPG data between the care-giver and participants is feasible and would increase the reliability of the therapeutic assessment.

### Supporting Information

Matlab codes are for the calculations of the three spectral-distortion measures and phylogenetic tree reconstruction of the PPG signals. Preprocessed finger pulse-wave data (3 minutes of recording) are synchronized PPG signals of the care-giver and 18 selected participants. The data also include the preprocessed finger PPG measurements (3 minutes of recording) of the 18 participants, recorded before and after the synchronized cognitive therapy.

## Supporting Information

S1 Dataset Matlab file (E_ds.mat) of the smoothed and detrended PPG data of the elderly during the therapy. (MAT)

S2 Dataset Matlab file (M_ds.mat) of the smoothed and detrended PPG data of the middle-aged care-giver during the therapy. (MAT)

S3 Dataset Matlab file (B_ds.mat) of the smoothed and detrended PPG data of the elderly before the therapy. (MAT)

S4 Dataset Matlab file (A_ds.mat) of the smoothed and detrended PPG data of the elderly after the therapy. (MAT)

S1 Code Matlab function (spdistance.m) that performs the calculations of the spectral distortion measures. (M)

S2 Code Matlab function (trees18.m) that loads the four datasets, calls other functions to calculate the distortions, and plots the 18 “phylogenetic” trees. (M)
